# Identifying molecular genetic features and oncogenic pathways of clear cell renal cell carcinoma through the anatomical (PADUA) scoring system

**DOI:** 10.18632/oncotarget.7129

**Published:** 2016-02-02

**Authors:** Hui Zhu, Haoyan Chen, Zhiqian Lin, Guohai Shi, Xiaozhu Lin, Zhiyuan Wu, Xia Zhang, Xi Zhang

**Affiliations:** ^1^ Department of Radiology, Fudan University Shanghai Cancer Center, Department of Oncology, Shanghai Medical College, Fudan University, Shanghai 200032, China; ^2^ State Key Laboratory for Oncogenes and Related Genes, Division of Gastroenterology and Hepatology, Renji Hospital, Shanghai Institute for Digestive Diseases, Shanghai Jiao-Tong University School of Medicine, Shanghai 200001, China; ^3^ Department of Radiology, Renji Hospital, Shanghai Jiao-Tong University School of Medicine, Shanghai 200001, China; ^4^ Department of Urology, Fudan University Shanghai Cancer Center, Department of Oncology, Shanghai Medical College, Fudan University, Shanghai 200032, China; ^5^ Department of Radiology, Ruijin Hospital, Shanghai Jiao-Tong University School of Medicine, Shanghai 200025, China; ^6^ Department of Oncology, East Hospital, Tongji University School of Medicine, Shanghai 200120, China; ^7^ Department of Physiology and Neurobiology, University of Connecticut, Storrs, CT 06269-3156, USA

**Keywords:** renal cell carcinoma, PADUA, gene mutation, miRNA subtypes, oncogenic pathways

## Abstract

Although the preoperative aspects and dimensions used for the PADUA scoring system were successfully applied in macroscopic clinical practice for renal tumor, the relevant molecular genetic basis remained unclear. To uncover meaningful correlations between the genetic aberrations and radiological features, we enrolled 112 patients with clear cell renal cell carcinoma (ccRCC) whose clinicopathological data, genomics data and CT data were obtained from The Cancer Genome Atlas (TCGA) and The Cancer Imaging Archive (TCIA). Overall PADUA score and several radiological features included in the PADUA system were assigned for each ccRCC. Despite having observed no significant association between the gene mutation frequency and the overall PADUA score, correlations between gene mutations and a few radiological features (tumor rim location and tumor size) were identified. A significant association between rim location and miRNA molecular subtypes was also observed. Survival analysis revealed that tumor size > 7 cm was significantly associated with poor survival. In addition, Gene Set Enrichment Analysis (GSEA) on mRNA expression revealed that the high PADUA score was related to numerous cancer-related networks, especially epithelial to mesenchymal transition (EMT) related pathways. This preliminary analysis of ccRCC revealed meaningful correlations between PADUA anatomical features and molecular basis including genomic aberrations and molecular subtypes.

## INTRODUCTION

The number of new cases of renal tumor in North America and Europe are the highest in the world [1]. According to the latest statistical data from National Cancer Institute, kidney and renal pelvis cancer incidence rates in the United States significantly rose from 7.6 per 100,000 people per year in 1975 to 15.28 in 2011 [2]. During 1999–2008, the majority of these cancer cases were renal cell carcinoma (RCC), which account for 94% and 93% of all kidney cancers among male and female, respectively [2]. Surgical treatments, as yet, are the only curative therapeutic approaches for early RCC. For example, partial nephrectomy (PN) is the gold standard treatment for localized kidney cancer [3]. Nephron-sparing surgery (NSS) is currently the most commonly performed surgical procedure for clinical T1 renal masses [4]. However, both PN and NSS have been underutilized due to their technical difficulty and surgical complexity [5]. Several nephrometry scoring systems (PADUA, R.E.N.A.L., C-index, DAP, etc.), which are all based on the anatomical features of renal tumors, have been developed to standardize preoperative evaluation, minimize the bias and predict the risk of complications [6–10].

Recently, some scholars introduced the field of “radiogenomics”, by integrating radiological and genetic methods as a novel diagnostic approach [11]. They found that radiological phenotypes, or “radiophenotypes”, with specific patterns of gene expression on a genome-wide scale can serve as a non-invasive surrogate to improve diagnostic classification and prediction of therapeutic response [12–15]. The PADUA scoring system is a standardized preoperative classification system developed to predict overall complications in patients with renal masses [6]. The classification of the PADUA score is based on tumor size and anatomical features of the tumor, including anterior or posterior face, longitudinal location, rim location, exophytic versus endophytic, and relationships with the renal sinus or urinary collecting system (UCS). Despite its importance, the molecular genetic basis underlying the PADUA scoring system is largely undetermined, which likely involves genetic aberrations and abnormal signaling pathways.

With the relatively recent advantages of high-throughput biological methods, genetic global profiling has led to better understanding of the pathological and clinical features of renal tumor. Loss-of-function and alterations of the von Hippel Lindau (VHL) tumor suppressor gene have been found in at least two-thirds of sporadic clear cell RCC (ccRCC) tumor tissue [16], and germline mutations involved in hereditary ccRCC as well [17]. Additionally, several key functional genes, such as MTOR, SETD2, KDM5C, PBRM1, PIK3C/A and BAP1, have been identified as significantly mutated genes (SMGs) in ccRCC [18]. Further study is expected to dissect out the individual role of these SMGs, or other molecular features, and integrate this information into clinical practice.

The use of the PADUA score can help clinicians stratify patients with different complication risks and select patients for NSS with different surgical approaches. To our knowledge, no study has been performed to elucidate the molecular genetic features and signaling pathways that related to the PADUA system. In this study, we applied our radiogenomics strategy to a gene mutation cohort study of ccRCC patients from The Cancer Genome Atlas (TCGA) database, in which we retrospectively acquired available CT images in The Cancer Image Archive (TCIA). The purpose of this study was to take advantage of the extensive molecular characterization, including somatic mutations, gene expression, miRNA molecular subtypes and oncogenic pathways, to summarize the molecular genetic basis that related to the PADUA scoring system and patient survival of ccRCC.

## RESULTS

### PADUA radiological features and gene mutation status

**Figure 1:**
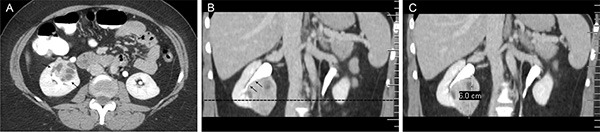
An example of PADUA radiological anatomical features analysis According to the PADUA scoring system, the following radiological anatomical features are evaluated in an axial contrast-enhanced CT image (**A**) and coronal multiplanar reformatted contrast-enhanced CT image (**B** and **C**). 1) Exophytic rate: a tumor that is < 50% exophytic is graded as 2 points (A and B); 2) Longitudinal location: a tumor crossing the sinus line (dotted line) > 50% is graded as 2 points (B); 3) Rim location: a tumor located at the medial rim is graded as 2 points (A and B); 4) Renal sinus: a tumor that involved the renal sinus (arrows in A) is graded as 2 points; 5) UCS: a tumor that involved the UCS (arrows in B) is graded as 2 points; 6) Tumor size: a 6.0 cm tumor is graded as 2 points (C); 7) Face location: a tumor on anterior faces of the kidney is indicated with a letter “a” (Figure A). Thus the total PADUA score of this case is 12a.

**Figure 2:**
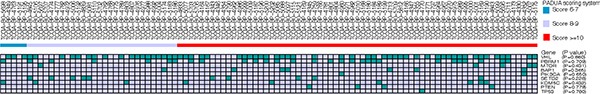
Association between PADUA overall score and gene mutation The gene mutation (green box) or wild type (white box) profile of low, intermediate and high PADUA score patients (labelled in blue, white and red bars) have been combined and summarized in a heatmap. The *P* values are calculated by Chi-square or Fisher's exact test.

The CT image traits evaluated in this study include tumor size and a few other anatomical parameters as previously integrated in the PADUA scoring system (Figure 1). The patients were stratified into three subgroups: low score (6–7), intermediate score (8–9) and high score (> 9) [6]. First, we investigated radiogenomic association of overall PADUA score and mutation frequency of 9 SMGs (VHL, PBRM1, SETD2, KDM5C, PTEN, BAP1, mTOR and TP53) in ccRCC [18] and no significant association was found (Figure 2). Next, we examined how the individual radiological features and gene mutation were related. Our results indicated a few strong correlations (Table 1). For example, mutation of mTOR was highly correlated with renal tumors that were located at the medial rim (PADUA score = 2, *p* = 0.008). Additionally, mutations of KDM5C and SETD2 were significantly associated with tumor size (*p* = 0.019 and *p* = 0.0445). KDM5C and SETD2 mutations were only detected in tumors smaller than 7 cm (PADUA score = 0 or 1). Interestingly, most of these two mutations (88.9% of KDM5C and 80.0% of SETD2) were only found in tumors smaller than 7 cm and bigger than 4 cm (PADUA score = 1). Analysis of TP53 and PTEN genes were not included because of lack of CT image data (less than five cases). These findings suggest that, at least in our cohort study, mutations of mTOR, KDM5C and SETD2 genes in renal tumors are specifically related to PADUA radiological features such as rim location and tumor size.

**Table 1:** Association of PADUA radiological features and gene mutations

	Longitudinal Location		Exophytic rate			Rim location		Renal sinus		UCS		Size			Face	
Point	1	2	1	2	3	1	2	1	2	1	2	1	2	3	A	P
BAP1																
*Mutant* (*n = 7*)	1	6	2	5	0	5	2	1	6	0	7	1	2	4	3	4
*Wild type* (*n = 93*)	23	70	39	47	7	56	37	31	62	15	78	28	43	22	49	44
*P* value	0.99		0.507			0.702		0.425		0.590		0.148			0.0.707	
KDM5C																
*Mutant* (*n = 9*)	3	6	4	5	0	5	4	2	7	3	6	1	8	0	3	6
*Wild type* (*n = 91*)	70	21	37	47	70	56	35	13	78	29	62	28	37	26	49	42
*P* value	0.781		0.689			0.733		0.621		0.99		0.019			0.305	
mTOR																
*Mutant* (*n = 5*)	0	5	1	3	1	0	5	1	4	1	4	1	2	2	3	2
*Wild type* (*n = 95*)	24	71	40	49	6	61	34	14	81	31	64	28	43	24	49	46
*P* value	0.333		0.386			0.008*		0.564		0.99		0.719			0.99	
PBRM1																
*Mutant* (*n = 29*)	11	18	9	18	2	18	11	3	26	7	22	6	15	8	18	11
*Wild type* (*n = 71*)	13	58	32	34	5	43	28	12	59	25	46	23	30	18	34	37
*P* value	0.069		0.417			0.99		0.543		0.349		0.559			0.270	
VHL																
*Mutant* (*n = 62*)	17	45	21	35	6	36	26	10	52	21	41	18	29	15	34	28
*Wild type* (*n = 38*)	7	31	20	17	1	25	13	5	33	11	27	11	16	11	18	20
*P* value	0.345		0.132			0.528		0.779		0.664		0.836			0.538	
SETD2																
*Mutant* (*n = 10*)	3	7	6	4	0	6	4	2	8	5	5	2	8	0	4	6
*Wild type* (*n = 90*)	21	69	35	48	7	55	35	13	77	27	63	27	37	26	48	42
*P* value	0.938		0.356			0.785		0.99		0.353		0.0445			0.641	

Note.

Abbreviations: UCS, urinary collection system.

Statistical method, Chi-Square Test or Fisher Exact test.

* Significant values.

### PADUA radiological features and miRNA molecular subtypes

**Figure 3:**
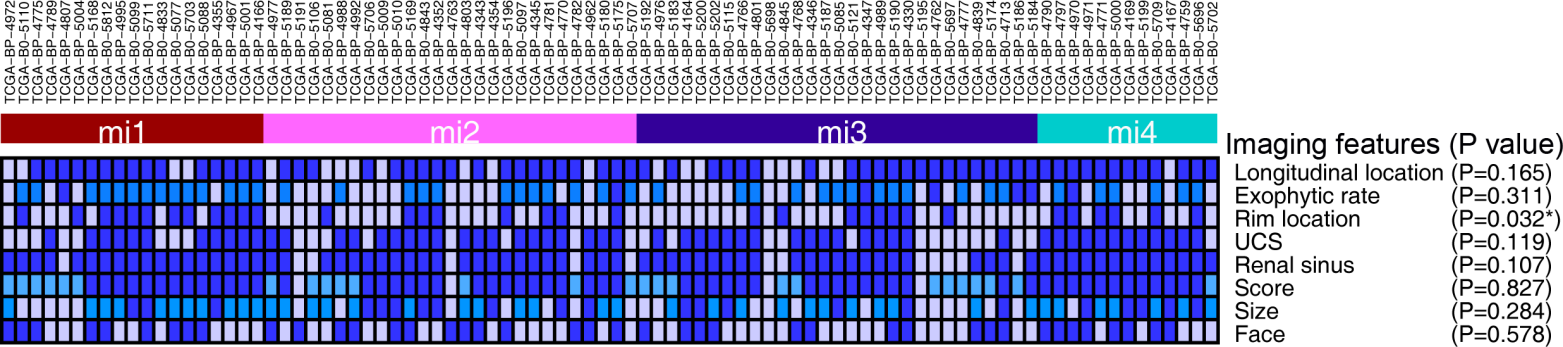
Association between PADUA radiological imaging features and miRNA molecular subtypes Value of individual PADUA radiological feature in four miRNA molecular subtypes is summarized in a heatmap. For each feature, white and dark blue (as well as light blue, if applicable) boxes indicate 1 and 2 (as well as 3, if applicable) points of PADUA grade, respectively. The *P* values are calculated by Chi-square or Fisher's exact test.

We also evaluated the correlation of the PADUA system and its radiological features with miRNA molecular subtypes [18]. Four stable clusters of miRNA subtypes (mi1–mi4) were previously identified by unsupervised clustering methods and correlated with different survival statuses [18]. miRNA clustering information was directly extracted from the online database. Although miRNA molecular subtypes have no significant correlation with the overall PADUA score, one miRNA subtype (mi1) is significantly enriched in the medial renal tumor (PADUA score = 2, *p* = 0.032, Figure 3). The mi1 subtype in renal tumors contains gene sets associated with chromatin remodeling processes. This particular association suggests that the medial rim location of renal tumors is correlated to the mi1 subtype and likely indicates the dysfunction of chromatin remodeling.

### PADUA radiological features and survival analysis

**Figure 4:**
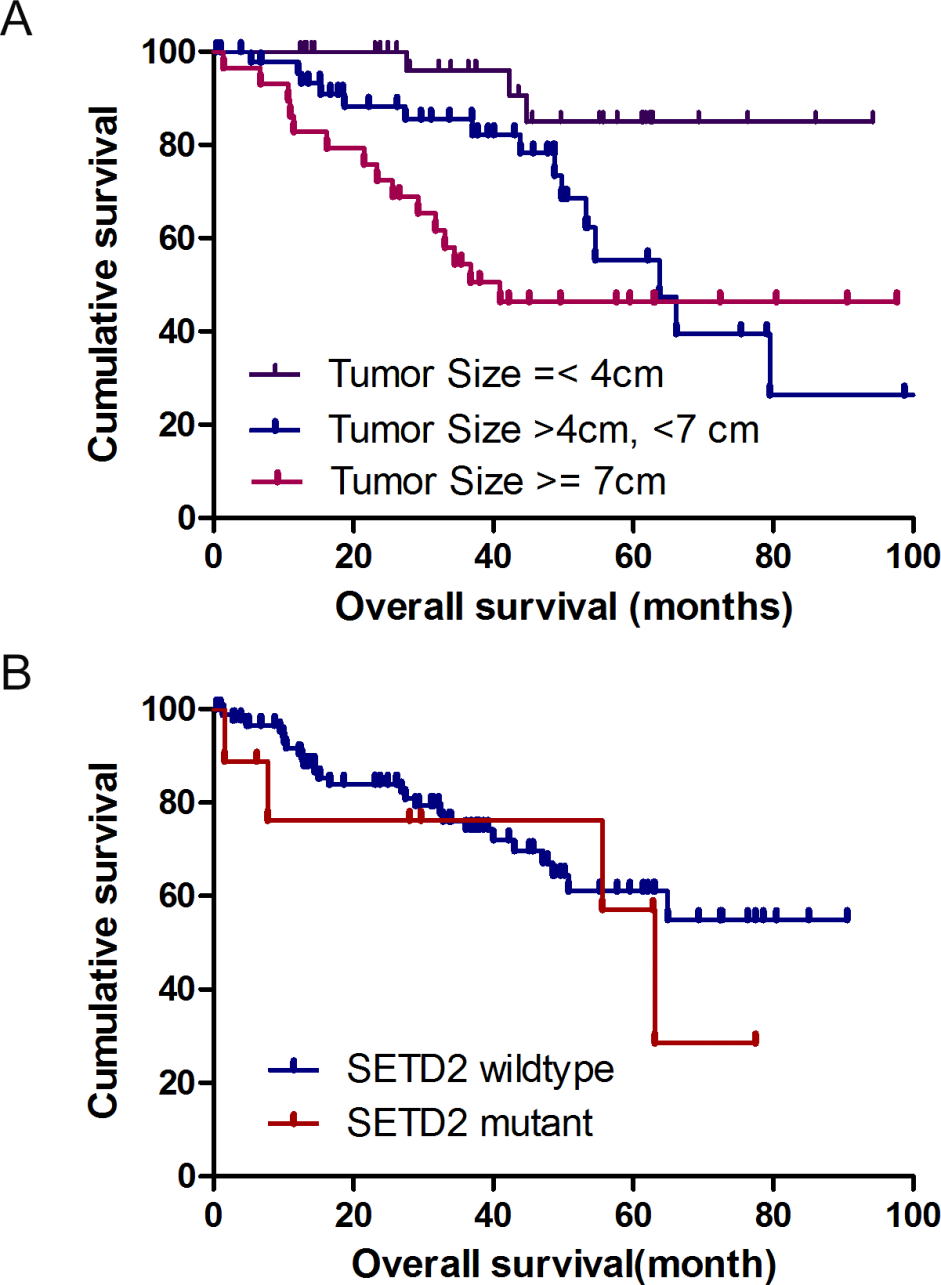
Kaplan-Meier survival analysis with respect to tumor size and gene mutations

Regarding the main hypothesis of our study, we tried to test if the PADUA system and its radiological features could be used for survival prediction. Even though overall survival was not significantly associated with the longitudinal location, exophytic rate, rim location, renal sinus, USC, face or total PADUA score (*p* > 0.05, data not shown); we did find that ccRCCs with a tumor size of > 7 cm was significantly associated with poor survival when compared to ccRCCs with a tumor size of < 4 cm (hazard ratio: 5.21, *p* < 0.01), and those with a tumor size of between 4 cm and 7 cm (hazard ratio: 2.68, *p* = 0.0159, Figure 4). Gene mutations (KDM5C and SETD2) that were highly correlated with ccRCC tumor size were not significantly correlated with survival (data not shown).

### PADUA scoring system and oncogenic pathways

**Figure 5:**
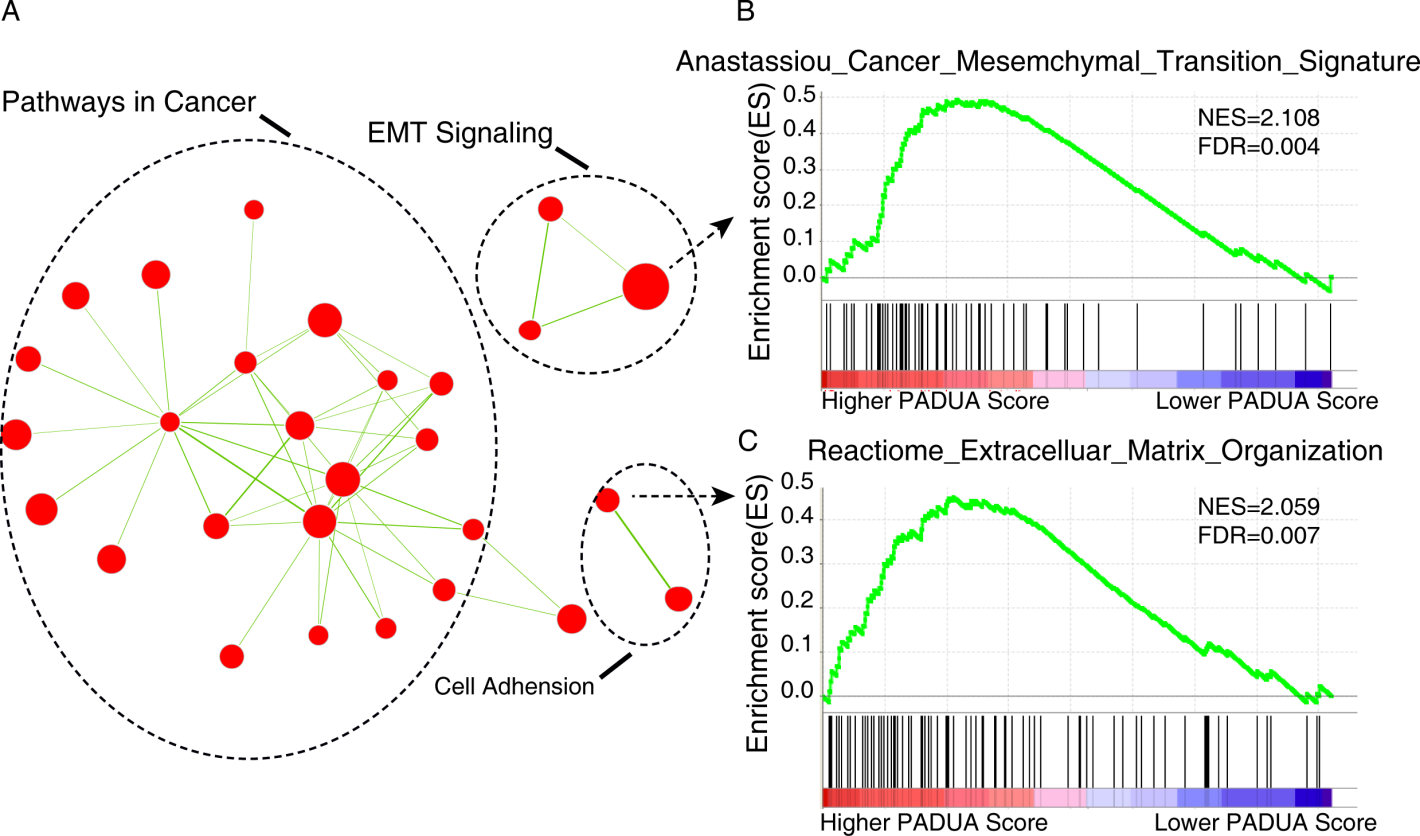
Gene set enrichment analysis (GSEA) delineates biological pathways and processes that correlations with PADUA score (**A**) Cytoscape and Enrichment Map are used for visualization of the GSEA results. Nodes represent enriched gene sets, which are grouped and annotated by their similarity according to related gene sets. Enrichment results are mapped as a network of gene sets (nodes). Node size is proportional to the total number of genes within each gene set. Proportion of shared genes between gene sets is represented by the thickness of the green line between nodes. This network map is manually curated removing general and uninformative sub-networks, resulting in a simplified network map shown in Figure 6A. Fully detailed GSEA results can be found in Table S1. (**B** and **C**) Enrichment plots are shown for a set of activated genes related to two epithelial to mesenchymal transition (EMT) related pathways: Anastassiou Cancer Mesenchymal Transition Signature (B) and Reactome Extracellular Matrix Organization (C). A positive value indicates more correlation with the samples with higher PADUA scores and a negative value indicates the opposite.

To probe the PADUA system's associated pathways, we performed GSEA to identify biological processes and signaling pathways correlating with the PADUA score. The genes were ranked from the patient with the highest PADUA score to the patient with the lowest score. Significant gene sets (FDR < 0.01, *p* < 0.01) were visualized as interaction networks with Cytoscape and Enrichment Map (Figure 5A and Supplementary Table S1). Interestingly, we found that the high PADUA score was related with numerous cancer-related networks. Notably, several epithelial to mesenchymal transition (EMT) related pathways (Anastassiou Cancer Mesenchymal Transition Signature (*p* < 0.001, FDR = 0.004), Jechlinger Epithelial to Mesenchymal Transition Up (*p* < 0.001, FDR = 0.143), Boquest Stem Cell Up (*p* < 0.001, FDR = 0.026) and Reactome Extracellular Matrix Organization (*p* < 0.001, FDR < 0.01)), were positively associated with a higher PADUA score (partly shown in Figure 5B and 5C).

## DISCUSSION

Previously, PADUA scores were considered one of the independent predictors of the occurrence of any grade complications [6]. To our best understanding, the current study elucidated the molecular genetic basis and oncogenic pathways that are significantly associated with the PADUA system and its radiological features in renal tumors.

KDM5C or SETD2 in ccRCCs have been associated with advanced stage, grade and possibly worse cancer-specific survival [19, 20]. Histone demethylase activity of KDM5C was required in the function of VHL in tumor growth [21]. Our study shows aberrations of KDM5C and SETD2 were majorly observed in medium-sized tumors (4–7 cm). This indicates these gene mutations may yield conflicting results in tumor growth. Lately, hyper-activated mutation of histone methyltransferase EZH2 has been identified in the pathogenesis lymphoma [22]. It is possible there are both activating and inactivating mutations in KDM5C and SETD2. Another possibility is both KDM5C and SETD2 may have broad substrate specificity, which may cause different effects on tumorigenesis [23]. We believe that further studies are needed to reveal the molecular characteristics of KDM5C and SETD2 mutations in RCC tumorigenesis.

Unsupervised clustering of miRNA expression data with a supervised learning method provides powerful strategies to identify molecularly and clinically significant cancer subtypes [24–27]. Out of four miRNA subtypes, mi1 subtype is the one with the longest survival time [18]. Here we found both mi1 miRNA subtypes and the mutation status of mTOR genes were significantly correlated with the medial rim location of renal tumors. Rim location is an essential feature of the PADUA scoring system that distinguishes PADUA from RENAL or C-index scoring systems. Thus, we can conclude that mutant mTOR and medial rim location are very likely correlated with mi1 subtype and longer survival time. Actually, mTOR is a well-validated signaling target in renal cancer carcinoma [16, 28]. A mutated PI3K/AKT/mTOR signaling pathway in ccRCC was identified in 2013 [29]. Currently mTOR is also being actively tested as a potential therapeutic target for cancers both pre-clinically and clinically [30, 31]. Interestingly, mTOR mutations lead to hyper-activation of mTOR pathway in ccRCCs [32]. Given the fact that our result shows ccRCC patients with mTOR mutation live longer than others, we predict hyper-activating mutated mTOR in ccRCC somehow inhibits tumorigenesis.

In the present study, we also found patients with higher PADUA scores were characterized by several EMT signaling pathways. EMT is an essential mechanism in embryonic development and wound healing [33]. Our study provides evidence for the meaningful underlying correlations between radiological anatomical features included in the PADUA scoring system and dysregulation of EMT signaling.

The current study will not only contribute to an improved characterization of ccRCC, but also provide a potential platform that allows PADUA scores and its radiological features to serve as molecular surrogates for ccRCC diagnosis, prognosis and personalized treatment for patients with specific genomic profiles. This study was limited to the number of patients carrying the mutant BAP1, KDM5C, mTOR, PBRM1 and VHL gene in the cohort study with CT images available. Additionally, this was not a prospectively designed study, and thus we were unable to truly examine the discriminatory power of these mutations.

## MATERIALS AND METHODS

### Patient population

The original clinicopathological and genomics data were obtained from TCGA while CT data were provided by TCIA. TCGA and TCIA are publicly available databases that contain no linkage to patient identifiers. All samples in TCGA have been collected and utilized under strict human subject protection guidelines, and institutional review board (IRB) approval. Originally, we obtained clinicopathological, genomics and CT images data of 123 ccRCC patients (October 1984 to January 1993) from TCGA and TCIA (Accessed on March, 2013). Each patient in TCIA could be matched within TCGA via a unique ID number. All patients must meet the following criteria to enter our study: available pathologic diagnosis of ccRCC from TCGA; available genomic data from TCGA; and available abdominal CT images from TCIA. 11 patients were excluded in our study due to the lack of data or low quality of data, and in total 112 patients were enrolled in our study. Thus, we identified all the patients with full annotations of age; gender; neoplasm histologic grade; TNM stage; tumor stage; mutation count; fraction of copy number altered genome; distant metastasis; mutations of genes of BAP1, KDM5C, mTOR, PBRM1, VHL, SETD2, mRNA and miRNA molecular subtype; and corresponding pretherapeutic CT images, which were available in TCIA.

### PADUA scoring system

According to the PADUA scoring system [6], the following radiological anatomical features and scores were assigned for each tumor. 1. Longitudinal location: The kidney was subdivided into upper, middle and lower parts by upper and lower renal sinus lines. A tumor being entirely above the upper or below the lower sinus line, or crossing the sinus line < 50%, was graded as 1 point, while a tumor crossing the sinus line > 50%, or being entirely between the sinus lines, was graded as 2 points. 2. Exophytic rate: Each tumor was classified into three groups: ≥ 50% exophytic (1 point), < 50% exophytic (2 points) and entirely endophytic (3 points). 3. Rim location: Each tumor was distinguished as being located at the lateral (1 point) or at the medial rim (2 points). 4. Renal sinus: Each tumor was divided into two types – tumor without renal sinus location (1 point) and tumor with renal sinus location or extension (2 points). 5. Urinary collection system (UCS): Each tumor was divided into two categories with respect to the UCS – absent relationship (1 point) and present relationship, namely, involving dislocation or infiltration of the UCS (2 points). 6. Tumor size: ≤ 4 cm (1 point), between 4 and 7 cm (2 points), and > 7 cm (3 points). 7. Face location: anterior (a) or posterior (p) faces were defined as those which were overlapped by the anterior or posterior layers of the renal fascia. They can be indicated with a letter “a” or “p” following the score (Figure 1). For each tumor, seven radiological anatomical features obtained six scores and one letter (a or p) according to the evaluation from three radiologists. Discrepancies between the evaluations of the three radiologists (H.Z., X.Z.L. and Z.Y.W) were minimal and were resolved by consensus.

### Genomic analysis

To explore the possibility that patients with different PADUA system scores have molecularly distinct tumors, we examined associations between the PADUA system score and several genomic features from tumors of 112 patients with ccRCC, represented both in TCGA and TCIA. Available genomic data included somatic mutation (*n* = 100), gene/mRNA expression (*n* = 97) and miRNA expression (*n* = 98). All genomic data were downloaded through the TCGA data portal (http://tcga-data.nci.nih.gov/tcga/findArchives.htm) and linked with patient characteristics. The molecular subtypes based on miRNA expression of samples were directly extracted from the online database [18].

Gene Set Enrichment Analysis (GSEA) was performed by the JAVA program (http://www.broadinstitute.org/gsea) using MSigDB C2 curated gene set collection. In GSEA, the PADUA score was considered as a continuous phenotype label. A Pearson metric was applied to rank genes and gene sets enriched at a nominal *P* < 0.05 and a false discovery rate < 0.25, as discussed by Subramanian et al. [34]. The GSEA outputs, visualized in Cytoscape (version 2.8.2) and the Enrichment Map software [35], were used to identify the biological processes associated with the PADUA score. To simplify the network map, a stringent threshold of gene-set permutations with a FDR cutoff of 0.5% and *p*-value cutoff of 0.01 was used in the Enrichment Map software as described [18]. The network map was manually curated, removing general and uninformative sub-networks and nodes.

### Image feature analysis

The CT images were presented to three radiologists (H.Z., X.Z.L. and Z.Y.W.) through a picture archiving and communication system (PACS, GE Healthcare Centricity RIS CE V2.0, GE Medical Systems, Fairfield, Connecticut, USA) for interpretation and measurement. Three radiologists, all with more than ten years of experience in interpreting abdominal CT images, reviewed the images in consensus according to predefined imaging trait definitions and exemplar images of the PADUA scoring system [6].

### Statistical analysis

Survival data, representing time between initial diagnosis and death, were downloaded directly from the TCGA data portal (http://tcga-data.nci.nih.gov/tcga/tcgaHome2.jsp). The correlations between survival, mutation status, molecular subtype and radiological anatomical features were analyzed separately. The analyses of radiological features and distribution of each mutation status (BAP1, KDM5C, mTOR, PBRM1 VHL and SETD2) and miRNA molecular subtypes were performed using Chi-square test or Fisher's Exact test. Finally, Kaplan-Meier method and log-rank test were used to compute overall median survival. All analyses of survival data were conducted by using the R program (www.r-project.org).

## CONCLUSIONS

In summary, this preliminary radiogenomics analysis of ccRCC identified several meaningful molecular genetic features correlated with radiological features included in the PADUA system. Our present study also associated the PADUA scoring system with EMT signaling pathways. Overall, our findings will facilitate the value of the PADUA scoring system in advancing individualized treatment. Future radiogenomics and functional studies are necessary to furnish more molecular evidence to strengthen the diagnostic implication of scoring systems.

## SUPPLEMENTARY MATERIALS TABLE




