# Supplementary material for: Identifying molecular genetic features and oncogenic pathways of clear cell renal cell carcinoma through the anatomical (PADUA) scoring system
Source: Oncotarget. 2016 Feb 2;7(9):10006–14. doi: 10.18632/oncotarget.7129 (PMC4891099; doi:10.18632/oncotarget.7129)
Supplement: Supplementary file 1 [file oncotarget-07-10006-s001.pdf]

Identifying molecular genetic features and oncogenic pathways of clear cell renal cell carcinoma through the anatomical (PADUA) scoring system

Supplementary Materials

Supplementary Table S1: Gene sets positively correlations with PADUA score
